# A Co-infection Model System and the Use of Chimeric Proteins to Study *Chlamydia* Inclusion Proteins Interaction

**DOI:** 10.3389/fcimb.2017.00079

**Published:** 2017-03-14

**Authors:** Ying Han, Isabelle Derré

**Affiliations:** ^1^Department of Molecular Biophysics and Biochemistry, Yale UniversityNew Haven, CT, USA; ^2^Department of Microbiology, Immunology and Cancer Biology, University of VirginiaCharlottesville, VA, USA

**Keywords:** *Chlamydia*, inclusion membrane protein, IncD, IncE, chimeric Inc protein, homo- and heterotypic interaction

## Abstract

*Chlamydia trachomatis* is an obligate intracellular bacterium associated with trachoma and sexually transmitted diseases. During its intracellular developmental cycle, *Chlamydia* resides in a membrane bound compartment called the inclusion. A subset of Type III secreted effectors, the inclusion membrane proteins (Inc), are inserted into the inclusion membrane. Inc proteins are strategically positioned to promote inclusion interaction with host factors and organelles, a process required for bacterial replication, but little is known about Inc proteins function or host interacting partners. Moreover, it is unclear whether each Inc protein has a distinct function or if a subset of Inc proteins interacts with one another to perform their function. Here, we used IncD as a model to investigate Inc/Inc interaction in the context of Inc protein expression in *C. trachomatis*. We developed a co-infection model system to display different tagged Inc proteins on the surface of the same inclusion. We also designed chimeric Inc proteins to delineate domains important for interaction. We showed that IncD can self-interact and that the full-length protein is required for dimerization and/or oligomerization. Altogether our approach can be generalized to any Inc protein and will help to characterize the molecular mechanisms by which *Chlamydia* Inc proteins interact with themselves and/or host factors, eventually leading to a better understanding of *C. trachomatis* interaction with the mammalian host.

## Introduction

*Chlamydia trachomatis* is an obligate intracellular bacterial pathogen responsible for the most common preventable blindness from infectious origin and is the leading cause of sexually transmitted infection of bacterial origin (Schachter, 1999). The ocular and genital tract epithelia are the primary sites of infection. After entry, *C. trachomatis* resides in a membrane-bound compartment, called the inclusion (Moulder, 1991). Within the lumen of the inclusion, the bacteria undergo a complex developmental cycle alternating between infectious and replicative forms. During co-evolution with the mammalian host, the *C. trachomatis* genome was reduced to about 900 open reading frames (ORF) (Stephens et al., 1998) and *C. trachomatis* has evolved sophisticated mechanisms to hijack cellular organelles and manipulate cellular pathways to acquire essential nutrients (Elwell et al., 2016). Central to these processes is a bacterial Type III secretion system (T3SS), which allows for the translocation of bacterial effectors from the bacterial cytosol into the host cell (Mueller et al., 2014). One family of *C. trachomatis* T3SS effectors, are inserted into the inclusion membrane and referred to as the inclusion membrane proteins (Inc) (Dehoux et al., 2011; Lutter et al., 2012; Moore and Ouellette, 2014).

*Chlamydia* inclusion membrane proteins are characterized by a large bilobed hydrophobic domain containing at least 50 amino acids (Bannantine et al., 2000), and amino- and carboxy-terminal tails that are presumably exposed on the cytosolic surface of the inclusion membrane. Based on the presence of the bilobed hydrophobic domain, the *C. trachomatis* genome is predicted to encode up to 60 Inc proteins. The inclusion membrane localization of a large number of predicted *C. trachomatis* Inc proteins has been confirmed using primary antibodies (Li et al., 2008) or expression of tagged proteins from the bacteria (Weber et al., 2015). These Type III secreted effectors are unique to *Chlamydia* and are strategically positioned to mediate the interaction of the inclusion with cellular factors and organelles.

Only a few *C. trachomatis* Inc proteins have been assigned a host interacting partner and/or a function. IncA is involved in homotypic fusion of inclusions (Hackstadt et al., 1999). IncG interacts with 14-3-3ß (Scidmore and Hackstadt, 2001) and CT229 with Rab4 (Rzomp et al., 2006). IncD interacts with and recruits the ceramide transfer protein, CERT, to ER-inclusion membrane contact sites (Derre et al., 2011; Agaisse and Derre, 2014). CT228 interacts with the myosin phosphatase target subunit 1 MYPT1 and regulates the mechanisms by which *C. trachomatis* egresses from the host cell (Lutter et al., 2013). CT850 interacts with the dynein light chain DYNLT1 (Mital et al., 2015). InaC (CT813/CTL0184) mediates the recruitment of 14-3-3ß, 14-3-3ϵ, and ARF1 to the inclusion membrane and is involved in actin assembly and Golgi positioning around the inclusion (Kokes et al., 2015). IncE binds to the sorting nexins SNX5 and SNX6, and recruits these retromer complex components to the inclusion, resulting in inclusion membrane tubulation (Aeberhard et al., 2015; Mirrashidi et al., 2015). Mirrashidi et al. have also identified putative mammalian interacting partners for nearly 40 *C. trachomatis* Inc proteins (Mirrashidi et al., 2015). The study was performed with the Inc proteins overexpressed in mammalian cells, so these interactions remain to be validated during infection, but the study detected IncD/CERT and CT228/MYPT1 interactions, suggesting that this human/Inc interactome is a solid foundation to further investigate the function of *C. trachomatis* Inc proteins.

It is unclear whether each Inc protein has a distinct function or if a subset of Inc proteins can act in concert, potentially through direct interaction. Some Inc proteins are evenly distributed on the surface of the inclusion membrane, while others are concentrated in microdomains. This is best illustrated with IncB, Inc101, Inc222, and Inc850, which co-localize to discrete punctae of the inclusion membrane (Mital et al., 2010). Inc222 and Inc850 were shown to interact, suggesting that Inc proteins could form stable complexes with one another. The homo- or heterotypic interaction of Inc proteins was independently investigated using a bacterial two-hybrid system (Gauliard et al., 2015). Inc222 and Inc850 interaction was observed using this experimental set up, and the data also suggested that IncD interacted with itself. In addition, IncA, IncG, IncF, CT229 (CTL0481), CT058 (CTL0314), and CT222 (CTL0475) were identified as potential interacting partners of IncD. While the CT058- and CT222-IncD interactions were reciprocal, the IncA-, IncG-, IncF-, and CT229-IncD interactions were unidirectional with this subset of Inc being able to interact with IncD, but IncD did not interact with these proteins.

Here we have developed a system to test Inc/Inc homo- and heterotypic interactions in the context of *C. trachomatis* infection and to identify domains that support these interactions. Our system relies on the homotypic fusion properties of *C. trachomatis* inclusions, on the co-infection with *C. trachomatis* strains expressing Inc proteins fused to different tags and on the expression of chimeric Inc proteins.

## Materials and methods

### Ethics statement

All genetic manipulations and containment work were approved by the UVA Biosafety Committee and are in compliance with the section III-D-1-a of the National Institutes of Health guidelines for research involving recombinant DNA molecules.

### Cell lines and bacterial strains

HeLa cells were obtained from ATCC (CCL-2) and cultured at 37°C with 5% CO_2_ in DMEM high glucose (Invitrogen) supplemented with 10% heat inactivated FBS (Invitrogen). *C. trachomatis Lymphogranuloma venereum, Type II* were obtained from ATCC (L2/434/Bu VR-902B). *Chlamydia* propagation and infection were performed as previously described (Derre et al., 2007).

### Plasmid construction

Restriction enzymes and T4 DNA ligase were obtained from New England Biolabs (Ipswich, MA). PCR was performed using Herculase DNA polymerase (Stratagene). PCR primers were obtained from Integrated DNA Technologies. All the plasmids used in this study are derivatives of p2TK2-SW2 mCh(Gro) (Agaisse and Derre, 2014). They express mCherry under the control of the *groESL* operon promoter and terminator, the TetR repressor and the indicated Inc under the control of the *tetA* gene promoter and *incDEFG* operon terminator. The p2TK2-SW2 mCh(Gro) Tet IncD-, IncE-, and IncG-3xFLAG plasmids were described previously (Agaisse and Derre, 2014; Mirrashidi et al., 2015). p2TK2-SW2 mCh(Gro) Tet IncD-Myc, CTL0314-3xFLAG, CTL0475-3xFLAG, or IncD/IncE-3xFLAG chimera were constructed similarly using the primers listed in Supplementary Table S1.

### *C. trachomatis* transformation

Our calcium-based transformation protocol was adapted from Wang et al. (2011) and is described in Agaisse and Derre (2013).

### Immunoblotting

Protein samples were separated by SDS-PAGE and transferred to nitrocellulose membranes. The membranes were blocked for 1 h at room temperature in 1xPBS containing 0.05% Tween and 5% Fat-free milk. Primary and HRP-conjugated secondary antibobies were diluted in 1xPBS containing 0.05% Tween and 5% Fat-free milk and respectively incubated over-night at 4°C and 1 h at room temperature. Proteins were detected using the Amersham ECL western blotting detection reagent as per manufacturer recommendation and a Biorad ChemiDoc imaging system.

### Immunofluorescence and microscopy

At the indicated times, HeLa cells seeded onto glass coverslips were fixed for 30 min in PBS containing 4% paraformaldehyde. Immunostainings were performed at room temperature. Antibodies were diluted in PBS containing 0.1% BSA and 0.1% Triton X-100. Samples were washed with PBS and examined under an epifluorescence or spinning disc confocal microscope.

### Antibodies

The following primary antibodies were used: Mouse monoclonal anti-FLAG [1:1,000 (IF), 1:20,000 (WB), Sigma], mouse monoclonal anti-Myc [1:1,000 (IF), 1:1,000 (WB), Cell Signaling], rabbit polyclonal anti-Actin (1:1,000, Sigma), rabbit polyclonal anti-tRFP (1:2,000, Evrogen), and rabbit polyclonal anti-IncA (1:200, kind gift from T. Hackstadt, Rocky Mountain Laboratories). The following secondary antibodies were used: Peroxidase-conjugated goat anti-rabbit IgG (1:10,000, Jackson ImmunoResearch), peroxidase-conjugated goat anti-mouse IgG (1:10,000, Jackson ImmunoResearch), goat anti-mouse AlexaFluor 488 or 514 (IF: 1:1,000, Molecular Probes), and goat anti-rabbit Pacific Blue (IF: 1:1,000, Molecular Probes).

### Co-infection and immunofluorescence analysis of co-infected cells

Each strain was used at an MOI of 5 to ensure that each eukaryotic cell would receive at least one of each bacterium leading to a mixed bacterial population in each inclusion and the cellular localization of the 3xFLAG- and Myc-tagged Inc proteins was analyzed by immunofluorescence. In our original experimental set up, the samples were co-stained with a rabbit anti-FLAG and a mouse anti-Myc. However, the rabbit anti-FLAG led to uneven staining of the inclusions (even at higher concentration). Duplicate coverslips from the same co-infection were therefore stained with mouse anti-FLAG or mouse anti-Myc antibodies. Although not ideal, the MOI were carefully optimized so that, for each co-immunoprecipitation experiment, 100% of the inclusions were positive for both FLAG and Myc. The samples were not analyzed if it was not the case.

### Co-immunoprecipitation

8.10^5^ HeLa cells plated in 6-well tissue culture dishes and infected with the indicated *C. trachomatis* strains for 24 h were washed once with 1x PBS and lysed for 20 min in 300 μl of lysis buffer [20 mM Tris pH7.5, 150 mM NaCl, 2 mM EDTA, 1%Triton X-100, 1 mM PMSF and protease inhibitor cocktail (Roche)]. The lysates were centrifuge at 13,000 rpm for 10 min. An aliquot of the clarified lysate was collected (Lysate). The clarified lysates were incubated for 2 h in the presence of 10 μl of anti-FLAG M2 agarose beads (Sigma). The beads were washed three times (20 mM Tris pH7.5, 150 mM NaCl, 2 mM EDTA, 1%Triton X-100) and the bound proteins were eluted with 15 μl of elution buffer [20 mM Tris pH7.5, 150 mM NaCl, 2 mM EDTA, 100 μg/ml 3XFLAG peptide (Sigma)]. Ten microliters of the eluted fraction was collected (IP). All steps were conducted at 4°C.

### Reproducibility

Each experiment was performed at least three times. Representative results are shown.

## Results

To investigate IncD interaction with a subset of Inc proteins in the context of *C. trachomatis* infection, we have generated *C. trachomatis* strains co-expressing mCherry under a constitutive promoter and IncD-3xFLAG, IncD-Myc, IncE-3xFLAG, IncG-3xFLAG, CTL0314-3xFLAG (CT058), or CTL0475-3xFLAG (CT222) under the control of the anhydrotetracycline (aTc) inducible promoter. IncG, CTL0314 and CTL0475 were chosen based on their potential ability to interact with IncD (Gauliard et al., 2015) and IncE was included as a negative control.

**Figure 1:**
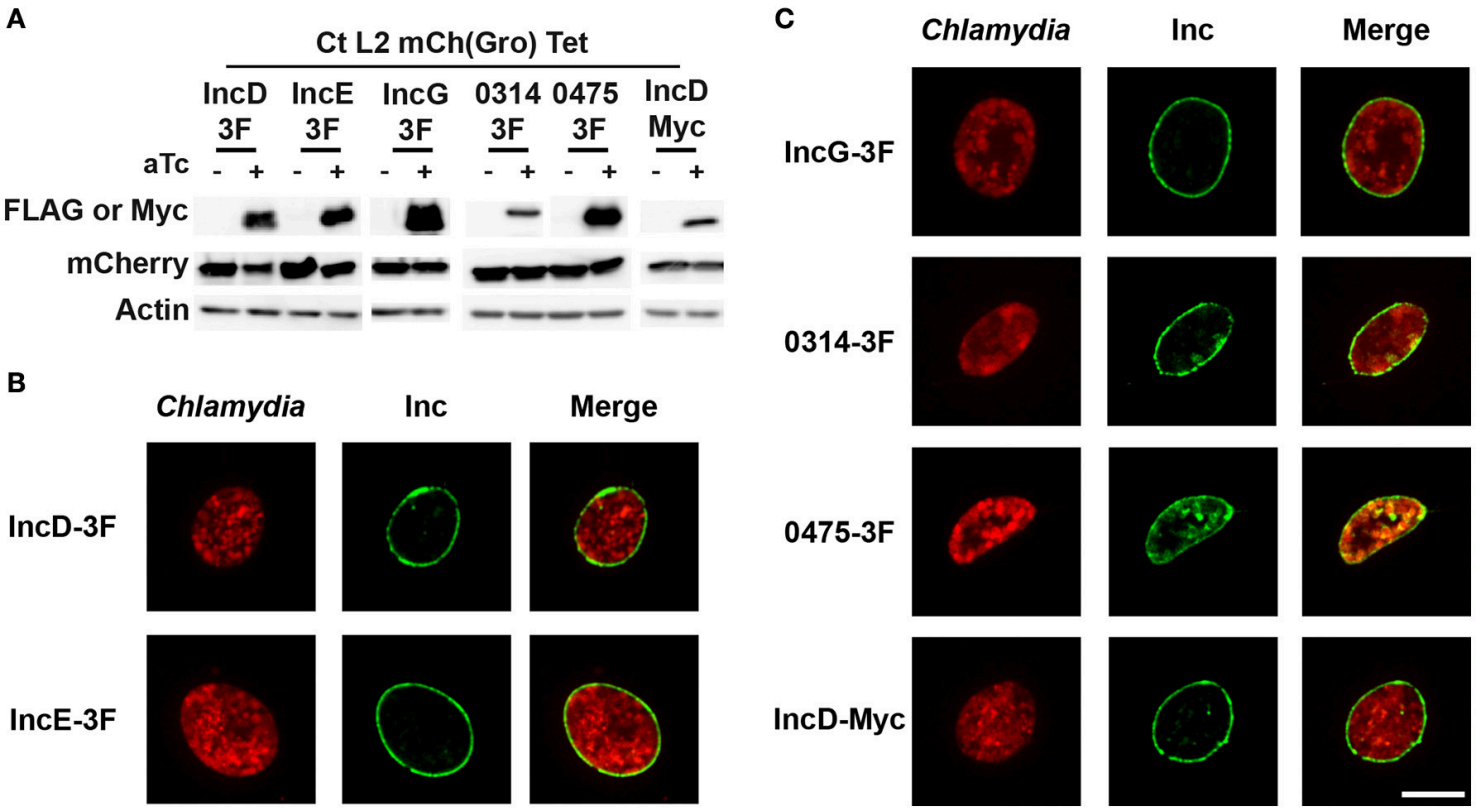
**Expression and inclusion localization of the subset of inclusion membrane proteins used in this study. (A)** Immuno-blot of cell lysates from HeLa cells infected with strains of *C. trachomatis* expressing mCherry constitutively and IncD-, IncE-, IncG-, CTL0314-, CTL0475-3xFLAG (3F), or IncD-Myc under the control of the aTc inducible promoter. The cells were infected for 24 h in the absence (-aTc) or in the presence (+aTc) of 2 ng/ml aTc and immuno-blot of the corresponding lysates were probed using antibodies against FLAG or Myc, mCherry, and Actin. **(B,C)** Confocal micrographs of inclusions of the *C. trachomatis* strains listed in **(A)**. The cells were infected in the presence of 2 ng/ml aTc, fixed 24 h post-infection, immunostained with anti-FLAG or anti-Myc antibodies and imaged using a confocal microscope. A single plane crossing the middle of the inclusion is shown. The left panels correspond to the bacteria (*Chlamydia*, red) and the middle panels to the 3xFLAG or Myc signal of the Inc constructs (Inc, green). The merge is shown on the right. Scale bar: 10 μm.

To verify the expression of the constructs, HeLa cells were infected with the above listed *C. trachomatis* strains in the absence or in the presence of aTc and the corresponding cell lysates were analyzed by western-blot (Figure 1A). Detection of actin and mCherry respectively confirmed equal cell and bacterial number in the absence (-aTc) or presence of aTc (+aTc). Anti-FLAG antibodies were used to detect the respective 3xFLAG tagged Inc proteins and anti-Myc antibodies were used to detect IncD-Myc. As expected, in the absence of inducer the proteins were not expressed (Figure 1A, -aTc), however a signal corresponding to the expected molecular weight of the respective proteins was observed in the presence of aTc (Figure 1A, +aTc).

In addition, the inclusion membrane localization of the above listed 3xFLAG- or Myc-tagged Inc proteins was analyzed by immuno-fluorescence. For this purpose, HeLa cells infected with the respective *Chlamydia* strains in the absence or in the presence of aTc were fixed 24 h post-infection and stained with antibodies against the FLAG or Myc tag. The respective Inc proteins were not detected in the absence of inducer (not shown). However, in the presence of aTc, 100% of the inclusions were positive for the respective constructs and the pattern indicated that, as previously shown for IncD-, IncE-, and IncG-3xFLAG (Agaisse and Derre, 2014; Mirrashidi et al., 2015), all constructs localized to the inclusion membrane (Figure 1B). The inclusion localization of CTL0314-3xFLAG and CTL0475-3xFLAG was further confirmed by co-staining of the inclusions with an anti-IncA antibody (Supplementary Figure 1).

**Figure 2:**
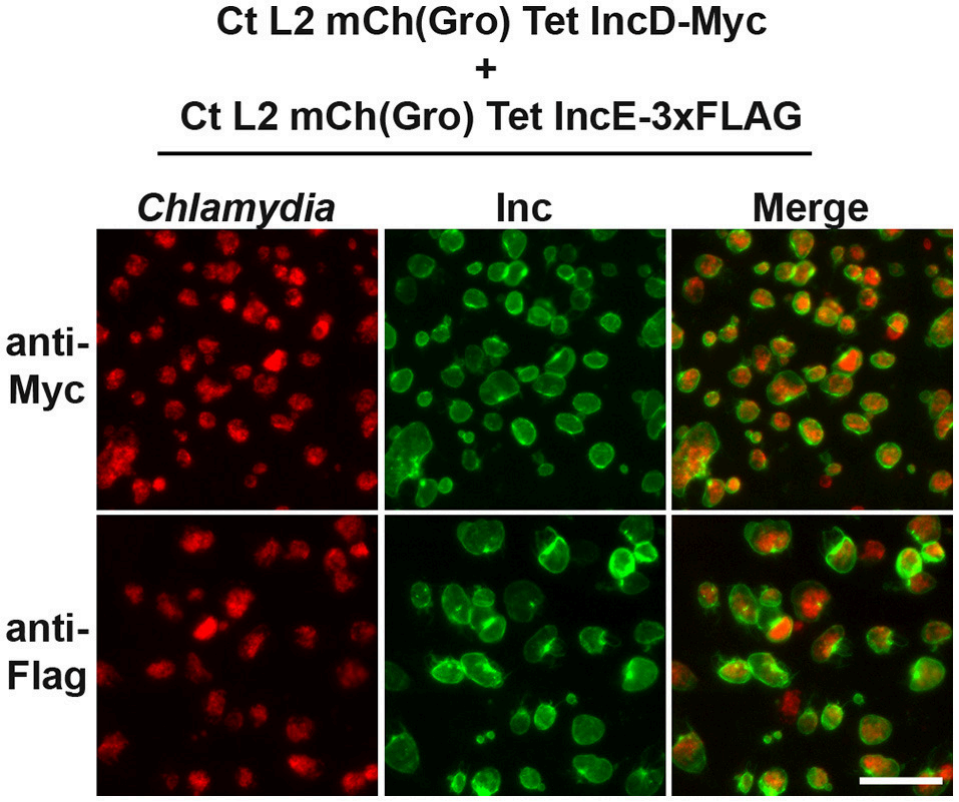
**Inclusion membrane localization of Inc proteins expressed by different ***C. trachomatis*** strains in a co-infection model system**. Epifluorescence micrographs of HeLa cells co-infected with strains of *C. trachomatis* expressing mCherry constitutively and IncE-3xFLAG or IncD-Myc under the control of the aTc inducible promoter. The cells were infected in the presence of 2 ng/ml aTc, fixed 24 h post-infection, immunostained with mouse anti-Myc antibodies (top panels) or mouse anti- FLAG antibodies (bottom panels) and imaged using an epifluorescence microscope. The left panels correspond to the bacteria (*Chlamydia*, red) and the middle panels to the Myc or FLAG signal of the Inc constructs (Inc, green). The merge is shown on the right. Scale bar: 50 μm.

To test the potential interaction between IncD and a subset of Inc proteins during *C. trachomatis* intracellular developmental cycle, we took advantage of the homotypic fusion properties of the *C. trachomatis* inclusions (Ridderhof and Barnes, 1989; Agaisse and Derre, 2013). HeLa cells were co-infected with two *C. trachomatis* strains expressing either IncE-3xFLAG or IncD-Myc. Each strain was used at an MOI of 5 to ensure that each eukaryotic cell would receive at least one of each bacterium leading to a mixed bacterial population in each inclusion. The cellular localization of the IncE-3xFLAG and IncD-Myc proteins was analyzed by immunofluorescence (Figure 2). Close to 100% of the inclusions were positive for FLAG (Figure 2, Top Panels) or Myc (Figure 2, Bottom Panels) when the samples were immuno-labeled with one or the other antibody. Altogether, this result confirmed that the co-infection method allows for the insertion of different Inc proteins into the same inclusion membrane when the proteins are produced by different strains.

**Figure 3:**
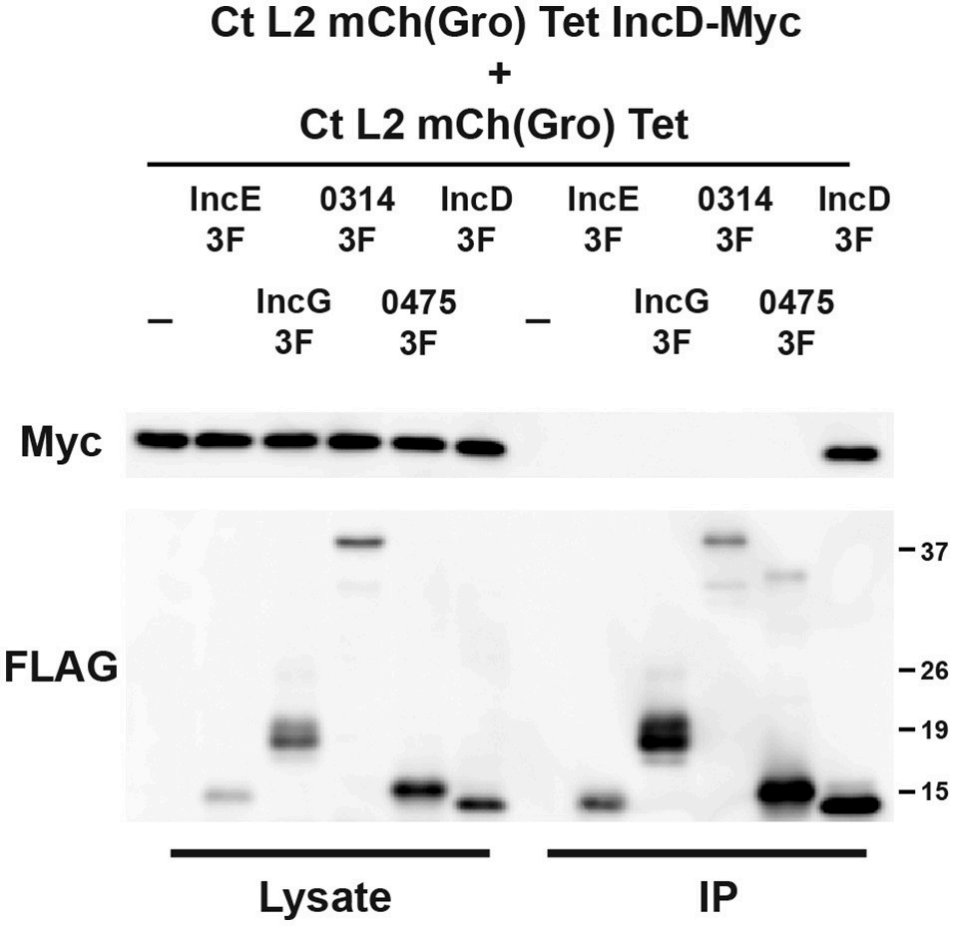
**IncD interacts with itself in the context of ***C. trachomatis*** infection**. Lysates from HeLa cells co-infected in the presence of 2 ng/ml aTc with a strain of *C. trachomatis* expressing mCherry constitutively and IncD-Myc under the control of the aTc inducible promoter [CtL2mCh(Gro)TetIncD-Myc] and a strain of *C. trachomatis* expressing mCherry constitutively and IncE-, IncG-, CTL0314-, CTL0475-, or IncD-3xFLAG (3F) under the control of the aTc inducible promoter [CtL2mCh(Gro)Tet] were immunoprecipitated with anti-FLAG M2 beads. A portion of the cell lysate (Left Panel, Lysate) and the immunoprecipitated proteins (Right Panel, IP) were separated by SDS-PAGE and analyzed by immunoblot (IB) with antibodies against Myc (Top Panels) and FLAG (Bottom Panels). The molecular weight ladder is shown on the right.

To investigate if IncD could interact with a subset of Inc proteins, including itself, HeLa cells were co-infected with the IncD-Myc expressing strain and a strain that expressed IncE-, IncG-, CTL0314-, CTL0475-, or IncD-3xFLAG, using the experimental set up described in Figure 2. For each condition, we confirmed that nearly 100% of the inclusions were positive for both markers (not shown). The samples were subjected to co-immuno-precipitation and the results were analyzed by western-blot (Figure 3). IncE-, IncG-, CTL0314-, CTL0475-, and IncD-3xFLAG were expressed and efficiently immuno-precipitated with the anti-FLAG conjugated beads. IncD-Myc did not co-immuno-precipitate with IncE-, IncG-, CTL0314-, or CTL0475-3xFLAG, suggesting that IncD does not interact with these Inc proteins. However, IncD-Myc did co-immuno-precipitate with IncD-3xFLAG (Figure 3, last lane), showing that IncD interacts with itself.

**Figure 4:**
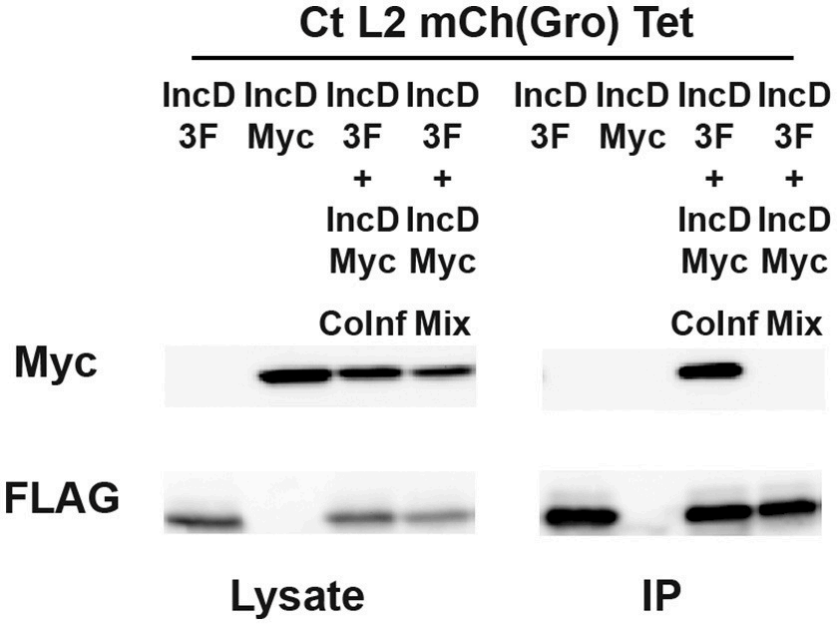
**IncD-IncD interaction occurs on ***C.trachomatis*** inclusion membrane**. HeLa cells were singly infected in the presence of 2 ng/ml with a strain of *C. trachomatis* expressing mCherry constitutively and IncD-3xFLAG (3F) or IncD-Myc under the control of the aTc inducible promoter or co-infected with these two strains (IncD-3F+IncDMyc CoInf). Lysates from singly and co-infected cells were collected and a fourth sample was prepared by mixing equal part of lysates from the singly infected cells (IncD-3F+IncDMyc Mix). The lysates were immunoprecipitated with anti-FLAG M2 beads. A portion of the cell lysate (Left Panel, Lysate) and the immunoprecipitated proteins (Right Panel, IP) were separated by SDS-PAGE and analyzed by immunoblot with antibodies against Myc (Top Panels) and FLAG (Bottom Panels).

We next investigated whether the IncD/IncD interaction was occurring when the proteins were inserted into the inclusion membrane or “*in vitro*” after the cell lysates were prepared and processed for immuno-precipitation. To address this question, we compared IncD self-interaction as described above, or after collecting and co-incubating cell lysates from cells that were singly infected with either the IncD-3xFLAG strain or the IncD-Myc strain. IncD self-interaction was observed upon co-infection (Figure 4, IP lane 3), however the IncD/IncD interaction was not detected when lysates from singly infected cells were combined prior immuno-precipitation (Figure 4, IP lane 4).

Altogether, these results indicate that, under our experimental set up, when *C. trachomatis* inclusion membrane proteins are inserted into the inclusion membrane, IncD interacts with itself but not with IncE, IncG, CTL0314, or CTL0475. Moreover, the IncD self-interaction was only observed when the IncD molecules were inserted into the same inclusion membrane.

We next sought to determine the IncD domain(s) mediating the IncD/IncD interaction. One possible approach would be to generate various internal, N- and C-terminal truncated variant of IncD and assay for their self-interaction. However, Inc proteins secretion through the *Chlamydia* type III secretion system requires a N-terminal secretion signal, that, if truncated, would prevent secretion. Moreover, the central hydrophobic domain, that is characteristic of Inc proteins, is most likely required for their insertion into the inclusion membrane, which would make its deletion incompatible with insertion of the corresponding Inc protein variant into the inclusion membrane.

**Figure 5:**
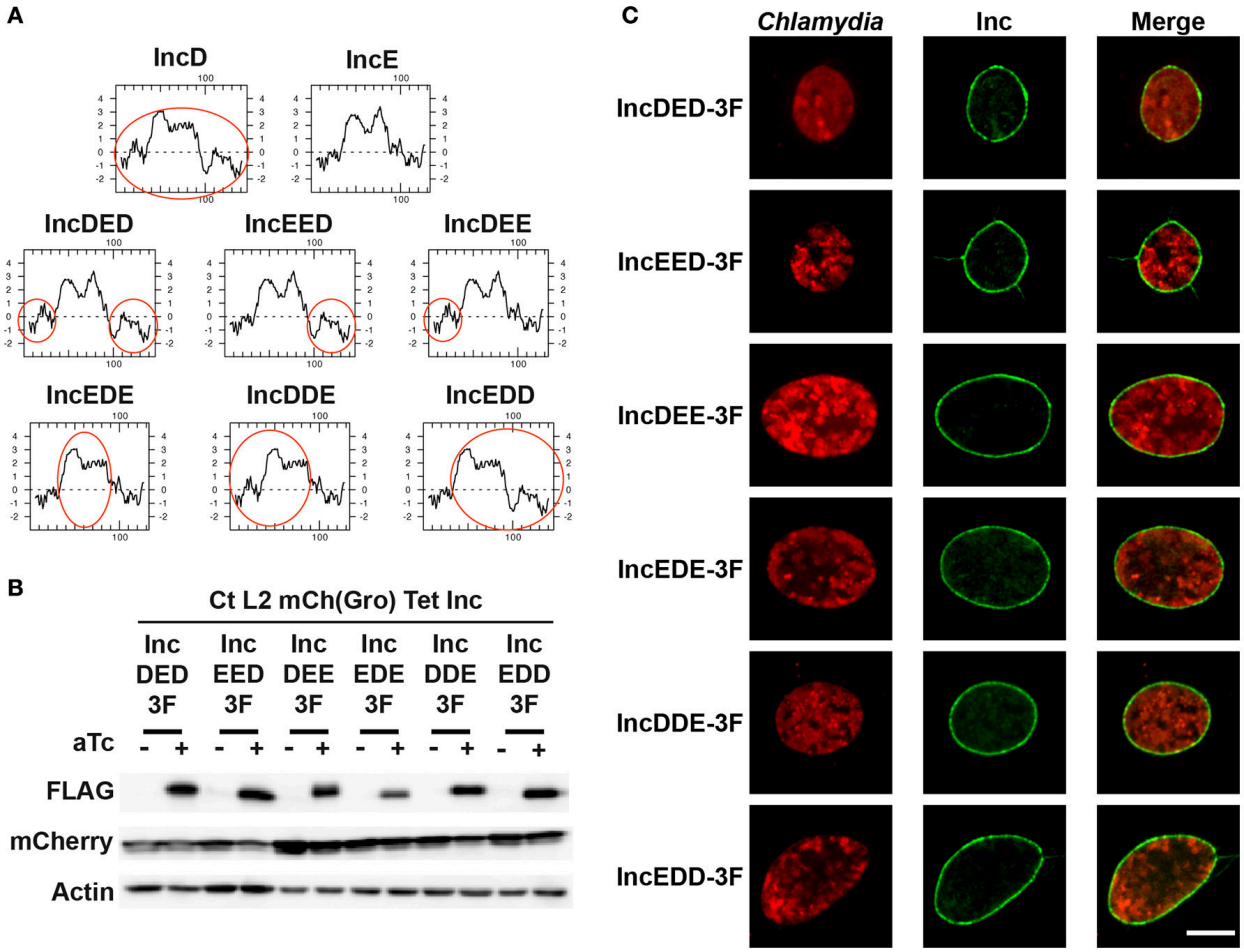
**IncD/IncE chimeric proteins localize to ***C. trachomatis*** inclusion membrane. (A)** Hydropathy plots of IncD, IncE, and the IncD/IncE chimeric proteins used in this study. The red circles indicate domains of IncD. **(B)** Immuno-blot of cell lysates from HeLa cells infected with strains of *C. trachomatis* expressing mCherry constitutively and the indicated IncD/IncE-3xFLAG (3F) chimeric proteins under the control of the aTc inducible promoter. The cells were infected for 24 h in the absence (-aTc) or in the presence (+aTc) of 2 ng/ml aTc and immuno-blot of the corresponding lysates were probed using antibodies against FLAG, mCherry, and Actin. **(C)** Confocal micrographs of inclusions of the *C. trachomatis* strains listed in **(B)**. The cells were infected in the presence of 2 ng/ml aTc, fixed 24 h post-infection, immunostained with anti-FLAG antibodies and imaged using a confocal microscope. A single plane crossing the middle of the inclusion is shown. The left panels correspond to the bacteria (*Chlamydia*, red) and the middle panels to the FLAG signal of the IncD/IncE chimera (Inc, green). The merge is shown on the right. Scale bar: 10 μm.

To address these issues, we generated chimeric proteins between IncD and IncE. IncE was chosen because it does not interact with IncD (Figure 3) and IncD and IncE have similar molecular weight and display similar hydropathy profiles (Figure 5A). Based on IncD and IncE respective hydropathy profiles, each protein was divided into three domains: The N-terminal domain (IncD: aa 1–39, IncE: aa 1–38), the hydrophobic domain (IncD: aa 40–91, IncE: aa 39–90), and the C-terminal domain (IncD: aa 92–146, IncE: aa 91–132). The following chimeric constructs were generated: IncDED, IncEED, IncDEE, IncEDE, IncDDE, and IncEDD where the first, second and third letter respectively correspond to the N-terminal, hydrophobic, or C-terminal domain of the respective Inc protein. The hydropathy profiles of the IncD/IncE chimeric proteins are shown in Figure 5A, where the IncD domains are indicated by the red circles. Each chimera was fused to a 3xFLAG tag and expressed under the control of the aTc inducible promoter into p2TK2-SW2mCh(Gro) and the corresponding plasmids were introduced into *C. trachomatis*. When HeLa cells were infected with the resulting *C. trachomatis* strains, mCherry was constitutively expressed and the IncD/IncE-3xFLAG chimeric proteins were only detected in the presence of inducer (Figure 5B). The levels of expression of the chimera were comparable to the one of IncD- or IncE-3xFLAG (Figure 1A) and the inclusion localization of the constructs was also confirmed by immunofluorescence (Figure 5C).

**Figure 6:**
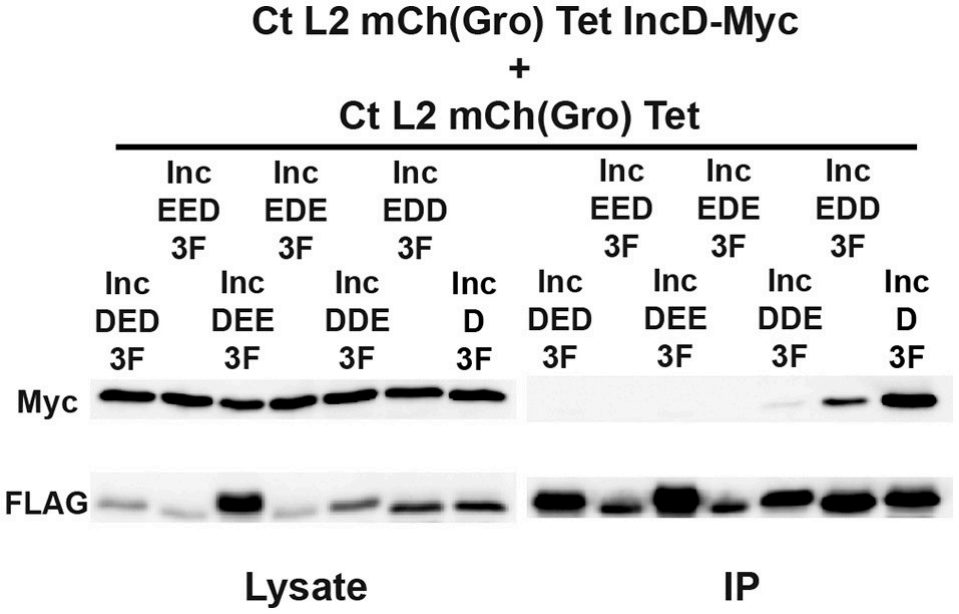
**IncD-IncD self-interaction requires full length IncD**. Lysates from HeLa cells co-infected in the presence of 2 ng/ml aTc with a strain of *C. trachomatis* expressing mCherry constitutively and IncD-Myc under the control of the aTc inducible promoter [CtL2mCh(Gro)TetIncD-Myc] and strain of *C. trachomatis* expressing mCherry constitutively and the indicated IncD/IncE-3xFLAG (3F) chimera under the control of the aTc inducible promoter [CtL2mCh(Gro)Tet] were immunoprecipitated with anti-FLAG M2 beads. A portion of the cell lysate (Left Panel, Lysate) and the immunoprecipitated proteins (Right Panel, IP) were separated by SDS-PAGE and analyzed by immunoblot (IB) with antibodies against Myc (Top Panels) and FLAG (Bottom Panels).

If the N-terminal, the hydrophobic, or the C-terminal domain of IncD is sufficient to mediate IncD self-interaction, we rationalized that this domain would promote IncD/IncD-E chimera interaction in our co-infection experimental set up. To test our hypothesis, HeLa cells were co-infected with *C. trachomatis* strains respectively expressing IncD-Myc and IncD-3xFLAG or IncD-Myc and one of the six IncD/IncE-3xFLAG chimeric proteins. For each co-infection combination, we confirmed that nearly 100% of the inclusions were positive for each construct by immuno-fluorescence (data not shown). The lysates were subjected to immuno-precipitation using anti-FLAG antibodies and co-immuno-precipitation of IncD-Myc was assayed by western-blot (Figure 6). The IncD/IncE-3xFLAG chimeric proteins were immuno-precipitated as efficiently as IncD-3xFLAG. As observed before (Figures 3, 4) IncD-Myc co-immuno-precipitated with IncD-3xFLAG. In addition, IncD-Myc co-immuno-precipitated with two of the IncD/IncE chimeric constructs, IncDDE and IncEDD, but the IncD/IncDDE and IncD/IncEDD interactions were not as robust as the one observed with IncD/IncD.

Altogether these results confirmed that chimeric Inc proteins could be engineered and successfully inserted into *C. trachomatis* inclusion membrane and could be used to dissect the molecular mechanisms driving Inc/Inc protein interaction. Our investigation of IncD self-interaction revealed that although IncD/IncE chimeric proteins containing the N-terminus and hydrophobic domain (DDE) or the hydrophobic domain and the C-terminus (EDD) of IncD were able to interact with IncD, it appears that the full length IncD protein is required for efficient self-interaction.

## Discussion

Inclusion membrane proteins are specific to *Chlamydia* and are strategically positioned to mediate inclusion interaction with host factors and organelles. However, little is known about Inc proteins interacting partners and/or function. Inc proteins are difficult to study because of their inherent biochemical properties. Their large hydrophobic domain complicates protein purification in a native and soluble state and limits subsequent *in vitro* studies to soluble domains (Ronzone and Paumet, 2013; Ronzone et al., 2014). Up until recently, in the absence of genetic tools to manipulate *Chlamydia*, studies have focused on over-expressing Inc proteins in mammalian cells (Rzomp et al., 2006; Derre et al., 2011; Mital et al., 2013; Mirrashidi et al., 2015) or using two-hybrid system in yeast (Scidmore and Hackstadt, 2001; Rzomp et al., 2006; Lutter et al., 2013; Mital et al., 2015) or bacteria (Gauliard et al., 2015). While these studies have led to the identification of Inc proteins interacting partners and shed light on the putative function of some Inc proteins, the big caveat of these studies is that the Inc proteins were not studied in the natural context of the inclusion membrane, potentially leading to interaction artifacts due to the absence of lipids and/or bacterial and host proteins that would normally constitute the natural environment surrounding the Inc proteins.

With the development of *Chlamydia* genetic tools (Wang et al., 2011; Sixt and Valdivia, 2016), it is now possible to express Inc proteins of interest from the bacteria and to study their roles in the context of the inclusion membrane (Agaisse and Derre, 2014; Bauler and Hackstadt, 2014; Kokes et al., 2015; Weber et al., 2015, 2016). We have previously used this method to further investigate IncD/CERT interaction in the context of the infection and to confirm the direct role of IncD in CERT recruitment to the inclusion membrane (Derre et al., 2011; Agaisse and Derre, 2014).

In the current study, we sought to follow up on a bacterial two-hybrid study suggesting that IncD may interact with itself and with a subset of Inc proteins (Gauliard et al., 2015). To probe for Inc/Inc interaction in the context of *C. trachomatis* infection, we took advantage of the fact that nascent inclusions originating from bacteria expressing two different Inc protein variants will eventually undergo fusion resulting in inclusions that display both Inc proteins on the surface of their membranes.

We were able to detect IncD/IncD interaction indicating that IncD may act as a dimer or an oligomer. Our data are also consistent with IncD self-interaction occurring only when the protein is inserted into the inclusion membrane. In the light of our previous data, showing that IncD interacts with the PH domain of CERT (Derre et al., 2011; Agaisse and Derre, 2014), one could envision that IncD dimerization or oligomerization may enhance the efficacy of CERT recruitment to the inclusion.

In our model system, we did not detect the IncD/CTL0314 and IncD/CTL0475 interactions previously observed in a bacterial two-hybrid system. There are two possible explanations to this discrepancy: (1) we failed to detect these interactions under our experimental set up or (2) the interactions observed using the bacterial two-hybrid system were not physiologically relevant. These results emphasize the need of validating any Inc/Inc interaction by different approaches, preferably in the context of infected cells.

If IncD self-interacts, what are the domains driving this interaction? IncD is a 15 kDa protein that can be separated into three major domains: A N-terminal domain (aa1–39) that contains the Type III secretion signal, a central hydrophobic domain (aa40–91) responsible for inclusion membrane anchoring and a C-terminal domain (aa92–146). Both the N- and C-terminal domains are predicted to face the cytosol. To study IncD variants, that are potentially defective for IncD/IncD interaction, in the context of the inclusion membrane, these variants should retain the Type III secretion signal and the hydrophobic domain for inclusion membrane localization.

Given that IncD is a fairly small protein, the type of truncated variants that can be generated and studied is therefore limited. We did generate a variant of IncD that lacked the C-terminal domain, but although the corresponding protein was expressed in *C. trachomatis*, it failed to display strong inclusion localization (data not shown).

We therefore turned to chimeric Inc proteins that contained various permutations of the N-, C-terminal, and hydrophobic domains of IncD and IncE, another small Inc protein that did not interact with IncD. Only two chimeras were able to interact with IncD. The combination of the N-terminal and hydrophobic domains of IncD (DDE) led to weak interaction and the hydrophobic domain combined to the C-terminal domain (EDD) led to a slightly more efficient binding. The efficacy of binding of these two chimeric proteins was however weaker than the one observed with the full-length protein. Our data suggest that the full-length IncD protein is required for dimerization and/or oligomerization. Alternatively, the hydrophobic domain may be sufficient to mediate IncD self-interaction but optimal self-interaction may require additional amino acids that were not included in our constructs, to accommodate optimal self-interaction. Finally, it is possible that, although the hydrophobic domain is sufficient to mediate IncD self-interaction, oligomers formed less efficiently between un-identical IncD units. Testing the self-interaction of the IncDDE and IncEDD chimera could address this question.

Our data suggest that, if not the full-length protein, a large portion of IncD is required for self-interaction and potentially for function. This would be in contrast with IncE, which has been proposed to be a monomer (Gauliard et al., 2015) and for which the C-terminal domain is sufficient for binding the PX domain of SNX5 and SNX6 (Mirrashidi et al., 2015). Altogether, one could envision that some Inc proteins, such as IncE, act as monomers and have distinct domains dedicated to interaction with host factors, while others, like IncD, require oligomerization of the full-length protein to efficiently recruit their host target to the inclusion membrane.

With the recent advances in *Chlamydia* genetics, it is now possible to investigate the role of *C. trachomatis* inclusion membrane proteins in the context of an infected cell. The co-infection system described here and the use of chimeric Inc proteins, together with the generation of *C. trachomatis* strains lacking Inc proteins of interest will be powerful tools to assay not only Inc protein functions, but also the molecular mechanisms by which Inc proteins interact with themselves or with host factors, ultimately leading to a better understanding of *Chlamydia* life cycle and interaction with the mammalian host.

## Author contributions

YH: Performed the experiments, Analyzed the data. ID: Designed the experiments, Performed the experiments, Analyzed the data, Wrote the manuscript.

## Funding

NIH NIAID grant R01AI101441 to ID.

### Conflict of interest statement

The authors declare that the research was conducted in the absence of any commercial or financial relationships that could be construed as a potential conflict of interest.

## Supplementary material

Supplementary Figure 1 **Inclusion localization of CTL0314- and CTL0475-3xFLAG**. Confocal micrographs of inclusions of *C. trachomatis* strains expressing mCherry constitutively and CTL0314-3xFLAG (Top panels) or CTL0475-3xFLAG (Bottom panels) under the control of the aTc inducible promoter. The cells were infected in the presence of 2 ng/ml aTc, fixed 24 h post-infection, immunostained with anti-FLAG (Yellow), and anti-IncA (Blue) antibodies and imaged using a confocal microscope. A single plane crossing the middle of the inclusion is shown. The left panels correspond to the bacteria (*Chlamydia*, red). The merge is shown on the right. Scale bar: 10 μm.

Supplementary Table 1 **Primers used in this study**.
