# Supplementary figures and images for: A Co-infection Model System and the Use of Chimeric Proteins to Study Chlamydia Inclusion Proteins Interaction
Source: Front Cell Infect Microbiol. 2017 Mar 14;7:79. doi: 10.3389/fcimb.2017.00079 (PMC5348484; doi:10.3389/fcimb.2017.00079)

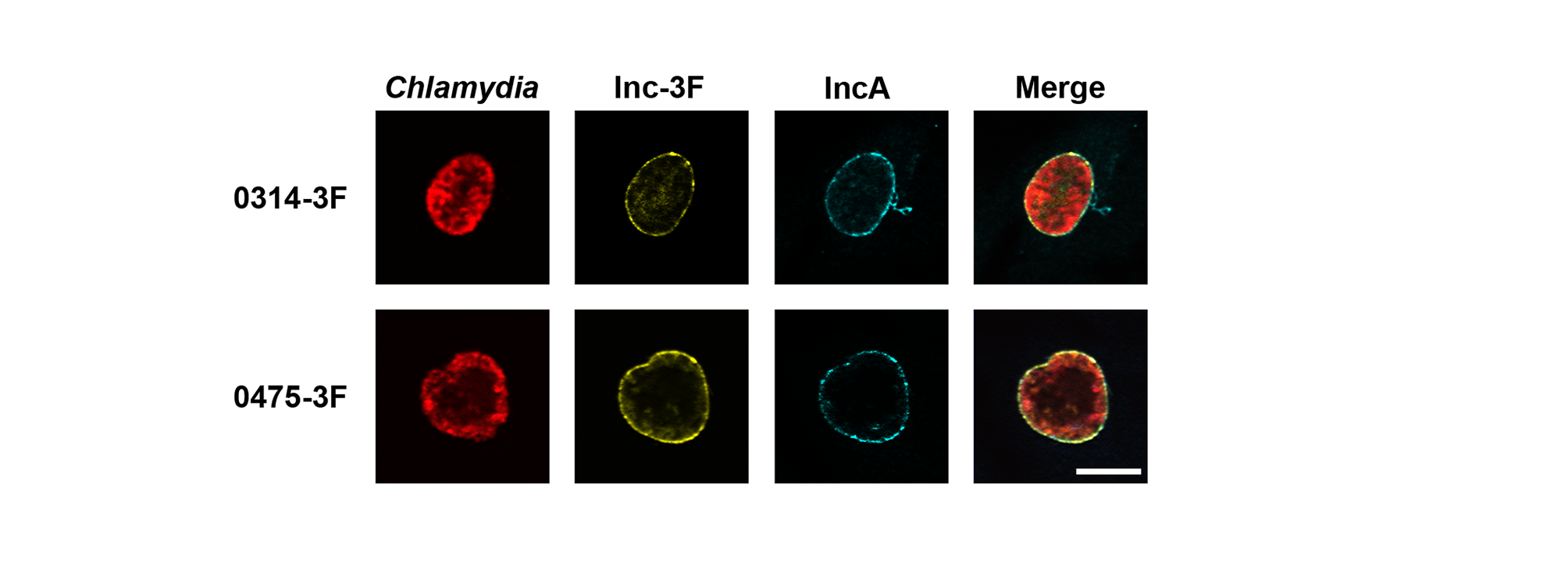

Supplement: Supplementary Figure 1 — Inclusion localization of CTL0314- and CTL0475-3xFLAG. Confocal micrographs of inclusions of C. trachomatis strains expressing mCherry constitutively and CTL0314-3xFLAG (Top panels) or CTL0475-3xFLAG (Bottom panels) under the control of the aTc inducible promoter. The cells were infected in the presence of 2 ng/ml aTc, fixed 24 h post-infection, immunostained with anti-FLAG (Yellow), and anti-IncA (Blue) antibodies and imaged using a confocal microscope. A single plane crossing the middle of the inclusion is shown. The left panels correspond to the bacteria (Chlamydia, red). The merge is shown on the right. Scale bar: 10 μm. [file Image1.TIF]
