# Supplementary material for: A Co-infection Model System and the Use of Chimeric Proteins to Study Chlamydia Inclusion Proteins Interaction
Source: Front Cell Infect Microbiol. 2017 Mar 14;7:79. doi: 10.3389/fcimb.2017.00079 (PMC5348484; doi:10.3389/fcimb.2017.00079)
Supplement: Supplementary Table 1 — Primers used in this study. [file Table1.DOCX]

Supplementary Table 1: Primers used in this study.

| **p2TK2-SW2 mCh(Gro)**  **TetIncD-Myc** |  |
| --- | --- |
| TetR STOP 5 Kpn | GGTGGTACCTTAAGACCCACTTTCACATTTAAG |
| IncDTerm 3 Not | GCGGGCGGCCGCgtcttaggagctttttgcaatgc |
| IncD Myc 5 | CGGTGAGTAAAAAAGGGGCGAGCatgGAACAAAAACTCATCTCAGAAGAGGATCTGTAAggatgacatgtgattcgcgtagg |
| IncD Myc 3 | cctacgcgaatcacatgtcatccTTACAGATCCTCTTCTGAGATGAGTTTTTGTTCcatGCTCGCCCCTTTTTTACTCACCG |
| **p2TK2-SW2 mCh(Gro) TetCTL0314-3xFLAG** |  |
| TetR STOP 5 Kpn | GGTGGTACCTTAAGACCCACTTTCACATTTAAG |
| IncDTerm 3 Not | GCGGGCGGCCGCgtcttaggagctttttgcaatgc |
| Tet0314 5 | cagtgatagagaaaagtgaaATGTTTACATCGCTGTCCGC |
| Tet0314 3 | GCGGACAGCGATGTAAACATttcacttttctctatcactg |
| 0314FLAG 5 | TCCCTCAACCCGTGAATTTAatgGACTACAAAGACCATGAC |
| 0314FLAG 3 | GTCATGGTCTTTGTAGTCcatTAAATTCACGGGTTGAGGGA |
| **p2TK2-SW2 mCh(Gro) TetCTL0475-3xFLAG** |  |
| TetR STOP 5 Kpn | GGTGGTACCTTAAGACCCACTTTCACATTTAAG |
| IncDTerm 3 Not | GCGGGCGGCCGCgtcttaggagctttttgcaatgc |
| Tet0475 5 | cagtgatagagaaaagtgaaATGCGTTGCTGTTGTGTTCG |
| Tet0475 3 | CGAACACAACAGCAACGCATttcacttttctctatcactg |
| 0475FLAG 5 | AAGCAATTAGTGTATTCCACatgGACTACAAAGACCATGAC |
| 0475FLAG 3 | GTCATGGTCTTTGTAGTCcatGTGGAATACACTAATTGCTT |
| **p2TK2-SW2 mCh(Gro) TetIncDED-3xFLAG** |  |
| TetR STOP 5 Kpn | GGTGGTACCTTAAGACCCACTTTCACATTTAAG |
| IncDTerm 3 Not | GCGGGCGGCCGCgtcttaggagctttttgcaatgc |
| TetDNEH 5 | CAGCGAAAAGATTGGCGGCAtgctgtttaggagttgtttg |
| TetDNEH 3 | caaacaactcctaaacagcaTGCCGCCAATCTTTTCGCTG |
| TetEHDC 5 | catctgctttggatgttctaACTACAGAAGCTGTGACTAG |
| TetEHDC 3 | CTAGTCACAGCTTCTGTAGTtagaacatccaaagcagatg |
| **p2TK2-SW2 mCh(Gro) TetIncEED-3xFLAG** |  |
| TetR STOP 5 Kpn | GGTGGTACCTTAAGACCCACTTTCACATTTAAG |
| IncDTerm 3 Not | GCGGGCGGCCGCgtcttaggagctttttgcaatgc |
| TetEHDC 5 | catctgctttggatgttctaACTACAGAAGCTGTGACTAG |
| TetEHDC 3 | CTAGTCACAGCTTCTGTAGTtagaacatccaaagcagatg |
| TetR STOP 5 Kpn | GGTGGTACCTTAAGACCCACTTTCACATTTAAG |
| IncDTerm 3 Not | GCGGGCGGCCGCgtcttaggagctttttgcaatgc |
| **p2TK2-SW2 mCh(Gro) TetIncDEE-3xFLAG** |  |
| TetR STOP 5 Kpn | GGTGGTACCTTAAGACCCACTTTCACATTTAAG |
| IncDTerm 3 Not | GCGGGCGGCCGCgtcttaggagctttttgcaatgc |
| TetDNEH 5 | CAGCGAAAAGATTGGCGGCAtgctgtttaggagttgtttg |
| TetDNEH 3 | caaacaactcctaaacagcaTGCCGCCAATCTTTTCGCTG |
| TetR STOP 5 Kpn | GGTGGTACCTTAAGACCCACTTTCACATTTAAG |
| IncDTerm 3 Not | GCGGGCGGCCGCgtcttaggagctttttgcaatgc |
| **p2TK2-SW2 mCh(Gro) TetIncEDE-3xFLAG** |  |
| TetR STOP 5 Kpn | GGTGGTACCTTAAGACCCACTTTCACATTTAAG |
| IncDTerm 3 Not | GCGGGCGGCCGCgtcttaggagctttttgcaatgc |
| TetENDH 5 | GAAAAAGTTCAGCTAGCAGCAgttgctgtggccactatattgg |
| TetENDH 3 | ccaatatagtggccacagcaacTGCTGCTAGCTGAACTTTTTC |
| TetDHEC 5 | ctttggttggaggagtgctgGAGAATCATGGTTTGGTGGG |
| TetDHEC 3 | CCCACCAAACCATGATTCTCcagcactcctccaaccaaag |
| **p2TK2-SW2 mCh(Gro) TetIncDDE-3xFLAG** |  |
| TetR STOP 5 Kpn | GGTGGTACCTTAAGACCCACTTTCACATTTAAG |
| IncDTerm 3 Not | GCGGGCGGCCGCgtcttaggagctttttgcaatgc |
| TetDHEC 5 | ctttggttggaggagtgctgGAGAATCATGGTTTGGTGGG |
| TetDHEC 3 | CCCACCAAACCATGATTCTCcagcactcctccaaccaaag |
| **p2TK2-SW2 mCh(Gro) TetIncEDD-3xFLAG** |  |
| TetR STOP 5 Kpn | GGTGGTACCTTAAGACCCACTTTCACATTTAAG |
| IncDTerm 3 Not | GCGGGCGGCCGCgtcttaggagctttttgcaatgc |
| TetENDH 5 | GAAAAAGTTCAGCTAGCAGCAgttgctgtggccactatattgg |
| TetENDH 3 | ccaatatagtggccacagcaacTGCTGCTAGCTGAACTTTTTC |
